# Supplementary material for: Genomic profiling of 553 uncharacterized neurodevelopment patients reveals a high proportion of recessive pathogenic variant carriers in an outbred population
Source: Sci Rep. 2020 Jan 29;10:1413. doi: 10.1038/s41598-020-58101-8 (PMC6989631; doi:10.1038/s41598-020-58101-8)
Supplement: Supplementary file 1 — Supplementary information. [file 41598_2020_58101_MOESM1_ESM.pdf]

< Supplementary Information >

Genomic profiling of 553 uncharacterized neurodevelopment patients reveals a high proportion of recessive pathogenic variant carriers in an outbred population

Youngha Lee[1], Soojin Park[2], Jin Sook Lee[2,3,4], Soo Yeon Kim[2], Jaeso Cho[1], Yongjin Yoo[1], Sangmoon Lee[1], Taekyeong Yoo[1], Moses Lee[1], Jieun Seo[1], Jeongeun Lee[1,5], Jana Kneissl[1], Jean Lee[1], Hyoungseok Jeon[1], Eun Young Jeon[1], Sung Eun Hong[1], Eunha Kim[1], Hyuna Kim[2], Woo Joong Kim[2], Jon Soo Kim[6], Jung Min Ko[2], Anna Cho[7], Byung Chan Lim[2], Won Seop Kim[8], Murim Choi[1,2] & Jong-Hee Chae[2]

[1]Department of Biomedical Sciences, Seoul National University College of Medicine, Seoul, 03080, Republic of Korea. [2]Department of Pediatrics, Seoul National University College of Medicine, Seoul, 03080, Republic of Korea. [3]Department of Pediatrics, Gil Medical Center, Gachon University College of Medicine, Incheon, 21565, Republic of Korea. [4]Department of Genome Medicine and Science, Gil Medical Center, Gachon University College of Medicine, Incheon, 21565, Republic of Korea. [5]Interdisciplinary Program for Bioengineering, Graduate School, Seoul National Universty, Seoul, 03080, Republic of Korea. [6]Department of Pediatrics, Chungbuk National University Hospital, Cheongju, 28644, Republic of Korea. [7]Department of Pediatrics, Ewha Womans University School of Medicine, Seoul, 07804, Republic of Korea. [8]Department of Pediatrics, College of Medicine, Chungbuk National University, Cheongju, 28644, Republic of Korea.

Youngha Lee, Soojin Park and Jin Sook Lee contributed equally to this work.

Correspondence and requests for materials should be addressed to M.C. (email: murimchoi@snu.ac.kr) or J.H.C. (email: chaeped1@snu.ac.kr)

Contents

Notable vignettes 4

Supplementary Figures

Figure S1. Patients categorized by clinical diagnosis (n = 553 patients). 7

Figure S2. The age distribution of all patients when their symptom began and the distance between age of onset to WES analysis (*n* = 553 patients). 8

Figure S3. Straight-line distances from SNUCH. 9

Figure S4. Identification of an inherited deletion at 2p22.3 in the family with hereditary spastic paraplegia (HSP). 10

Figure S5. Pathogenic variants categorized by function (n = 262 variants). 12

Figure S6. No difference in the number of recessive variants for neurodevelopment-related gene sets between patients and controls. 13

Figure S7. Distribution of pLI values 14

Figure S8. Presentation and validation of cases that WES-based analysis altered clinical courses. 15

Figure S9. Anatomical components of each brain regions that were used for novel gene network analysis 17

Supplementary Tables

Table S1. List of copy number variations discovered in this study. 18

Table S2. List of known neurologic disorder associated genes. 21

Notable vignettes

WES-based diagnoses of 553 patients with neuro-developmental problems generated cases that were clinically meaningful for a number of reasons (Table 2).

***ST3GAL5* case: genetic elucidation of a case of compound symptoms**[1]

Two female siblings presented with a Rett syndrome-like phenotype, such as psychomotor retardation/regression, delayed speech, hand stereotypies with a loss of purposeful hand movements, and choreoathetosis. Genetic tests of *MECP2/CDKL5/FOXG1* and chromosomal microarray found no plausible candidates. GM3 synthase deficiency with *ST3GAL5* compound heterozygous variants was diagnosed through WES, which was confirmed by liquid chromatography-mass spectrometry analysis[1,2]. WES ended three years of an undiagnosed period and broadened the phenotypic spectrum of this rare neurometabolic disorder.

DYNC1H1 case: a case of evolving symptoms

An 8-month-old girl from healthy parents presented with global developmental delay and generalized hypotonia. Laboratory findings, MRIs, electromyography/nerve conduction studies, and genetic tests including *SMN1*, failed to identify her etiology. Although her cognitive function regression improved rapidly to near normal, a suspicious paraplegic gait developed from three years of age. WES revealed a novel *de novo* missense variant in *DYNC1H1*, encoding a subunit of the cytoplasmic dynein complex (Fig. S8a-d). This observation led us to conclude that the patient started displaying global DD, but was eventually diagnosed with SMALED (spinal muscular atrophy, lower extremity-predominant), mimicking hereditary spastic paraplegia. This

case poses a rare example, along with recent recognition that *DYNC1H1* carriers display complex HSP[3], which demonstrates the clinical utility for pediatric cases with phenotypic pleiotropy and symptom evolution.

***ASAH1* case: a case with corrected diagnosis**[4]

An 11-month-old girl from healthy parents was referred to our hospital for developmental delay with hypotonia, facial dysmorphism, congenital heart defects, and a sacral mass since birth. Karyotyping, metabolic screening, and chromosomal microarray revealed no abnormalities. Excision of the sacral mass led to pathologic diagnosis of epithelioid hemangioendothelioma, which required repeated operations and chemotherapy. However, joint contracture and multiple subcutaneous nodules appeared from 18 months of age. WES revealed compound heterozygous variants in *ASAH1*, associated with Farber disease. After the establishment of the correct molecular diagnosis at the age of four, additional electron microscopic findings of previously excised masses confirmed the pathognomonic findings[4].

***SLC2A1* case: a case where an effective treatment was given**

A 16-year-old boy from an otherwise a healthy family displayed an abnormal gait from around eight years of age that later developed into falls with dystonia, notably in the lower extremities, which was provoked mainly by exercise or stress. His motor development was delayed, although he had normal head circumference and cognition. WES revealed a *de novo* LoF variant in *SLC2A1*, encoding a glucose transporter (GLUT1)[5] (Fig. S8e-g). We also retrospectively noticed that he had developed episodes of staring spells from an age of four years, suggesting absence seizures, which disappeared after three years of antiepileptic medication. Cerebrospinal fluid

analysis confirmed a low glucose level (37 mg/dL, 36% of serum glucose, normal range > 40%). Based on this observation, a ketogenic diet was applied and completely changed the quality of his life, with a resulting near-absence of dystonia.

RAB11B case: a case of newly identified disease through international data sharing[6]

A 2-year-old boy was referred for evaluation of global developmental delay. He showed microcephaly and severe cognitive impairment with epilepsy but without evidence of regression. Notably, abnormal acanthotic skin lesions progressed from the face to the whole body. We identified a *de novo* variant in *RAB11B* and submitted this to GeneMatcher[7]. Soon, four cases with a similar neurodevelopmental phenotype were matched and we demonstrated that this gene causes intellectual disability and a distinctive brain phenotype[6].

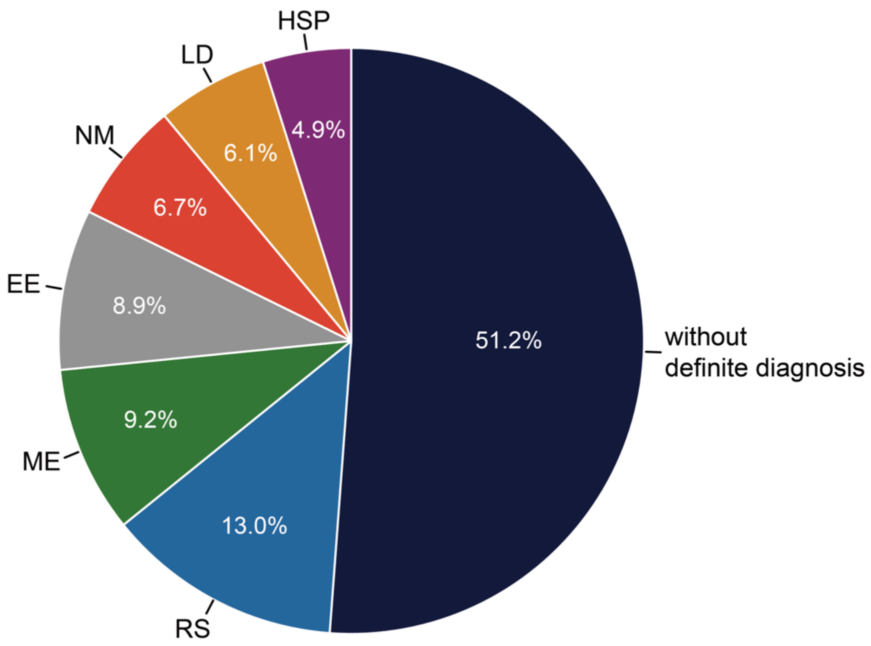

Figure S1. Patients categorized by clinical diagnosis (*n* = 553 patients). RS, Rett syndrome-like encephalopathy; ME, Mitochondrial encephalopathy; EE, Epileptic encephalopathy; NM, Neuromuscular disorder; LD, Leukodystrophy; HSP, Hereditary spastic paraplegia

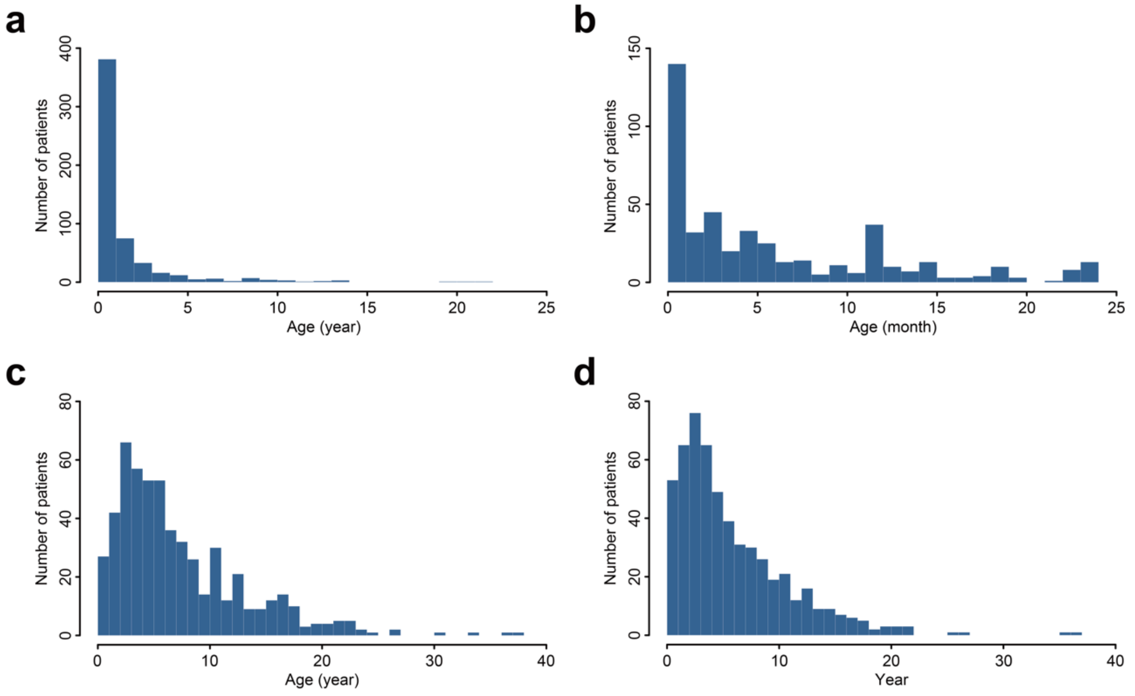

Figure S2. The age distribution of all patients when their symptom began and the time differences between age of onset to WES analysis (*n* = 553 patients). (a) The age of onset of all patients (year). (b) The age of onset of patients whose symptoms began before 24 months. (c) The Age distribution of all patients when the WES analysis was performed. (d) Distribution of time differences between patient's age of onset and the time of WES analysis.

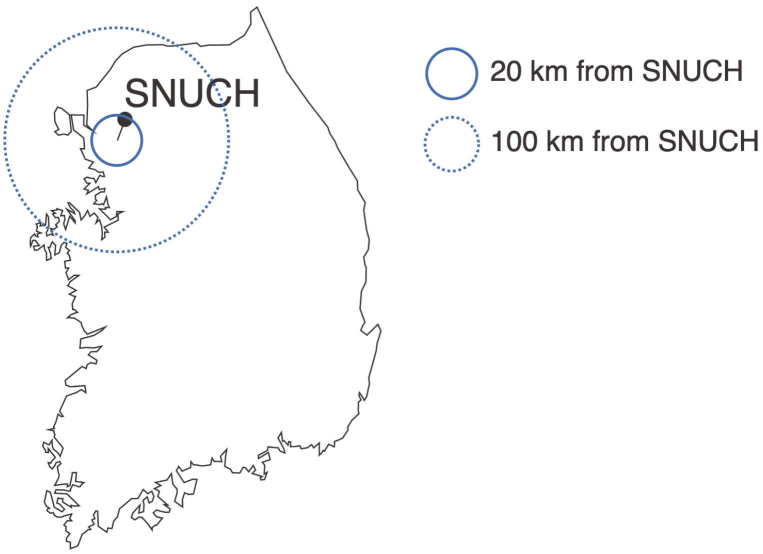

Figure S3. Straight-line distances from SNUCH. The 20 km radius circle includes most of Seoul, where about a quarter of entire population resides. The 100 km radius circle encompasses most of the Kyeonggi province, where another quarter of entire population resides.

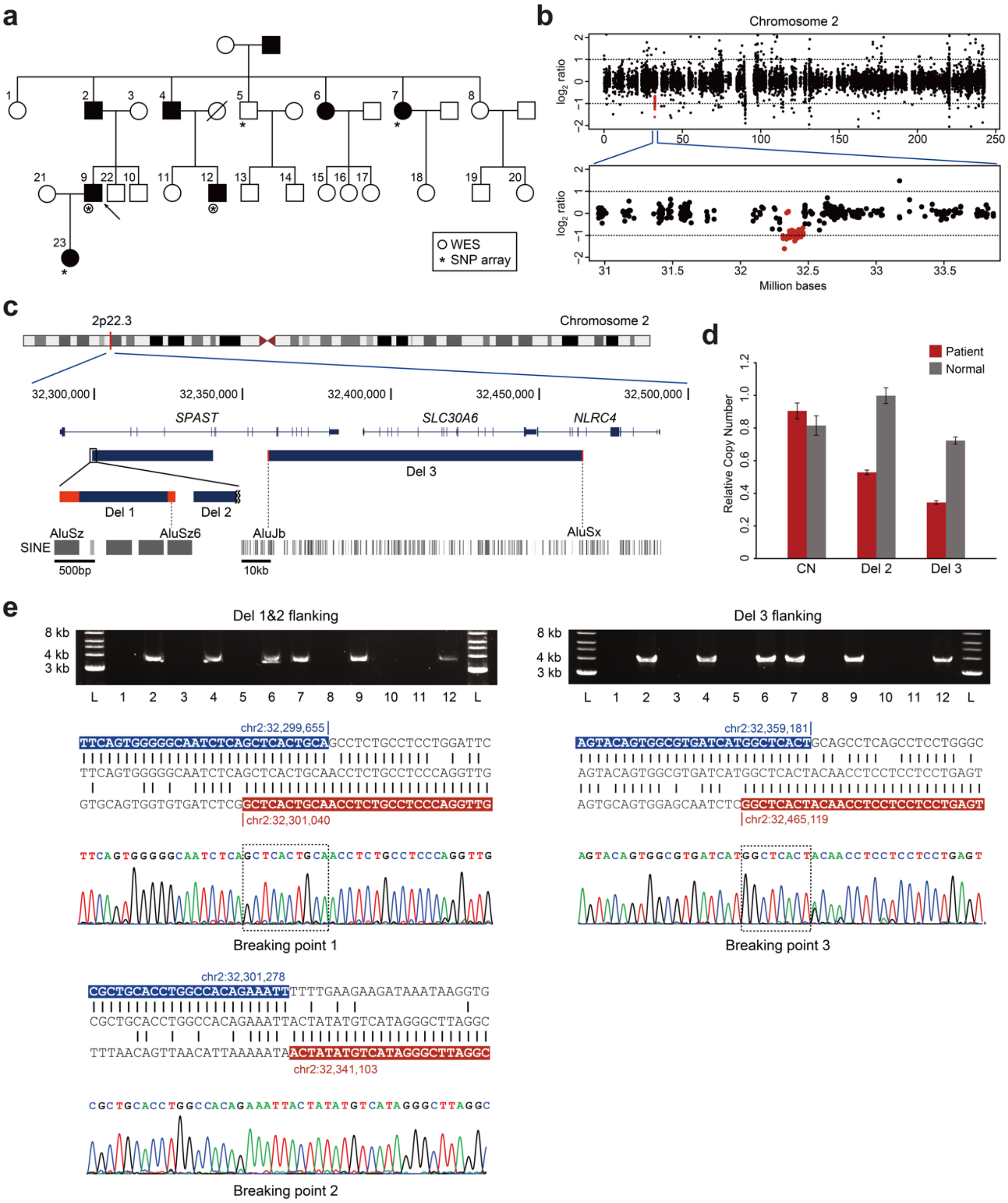

Figure S4. Identification of an inherited deletion at 2p22.3 in the family with hereditary spastic paraplegia (HSP). (a) Pedigree of an HSP family with 8 affected individuals across four generations. (b) WES-based log2-based copy-number values of subject HSP-9 compared with an unrelated normal subject on chromosome 2 showing the presence of heterozygous microdeletion. Captured intervals of copy-number loss are indicated by red dots. (c) Enlarged view of deletion-bearing region at 2p22.3

encompassing *SPAST*. Blue solid bars represent the deleted intervals. Alu repeat elements at the deletion breakpoints are indicated by red solid bars and aligned with the UCSC Genome Browser RepeatMasker, represented by dotted lines. (d) Validation of the deleted regions by quantitative PCR of genomic DNA. The red bars represent an average copy-number of five patients and the gray bars represent an average copy-number of five normal individuals from the family. Error bars represent standard error. CN: copy-neutral region. (e) DNA sequence analysis of the deleted regions. The DNA fragments containing the deletion was amplified by the deletion-specific primer pairs. The deletion-specific PCR products of 3.8 kb (Del 1 & 2) and 4.0 kb (Del 3) are observed in each affected individual. Reference sequences surrounding the breaking points are indicated in blue and red color.

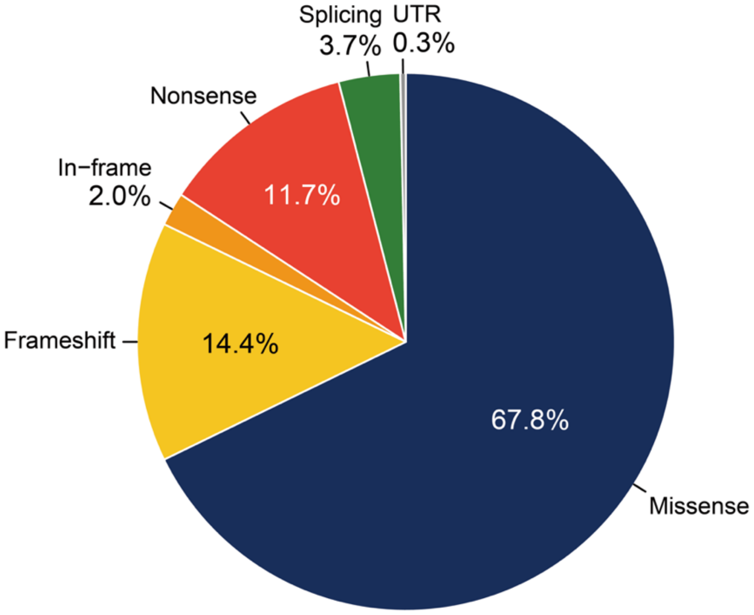

Figure S5. Pathogenic variants categorized by function (*n* = 298 variants).

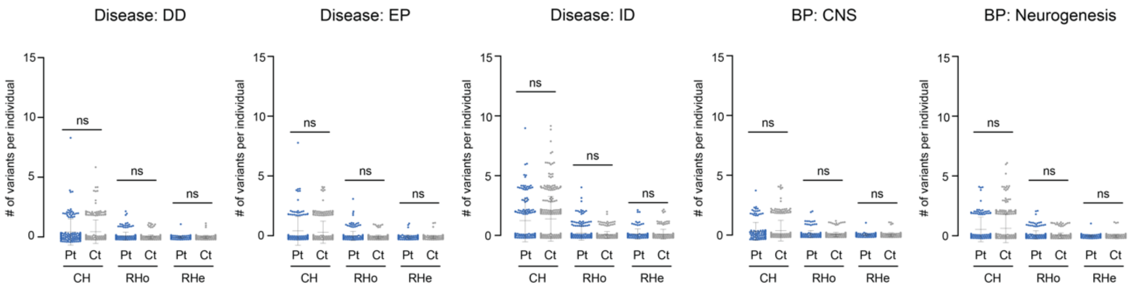

Figure S6. No difference in the number of recessive variants for neurodevelopment-related gene sets between patients and controls. All variants from genes from Disease: Developmental delay (DD), Disease: Epilepsy (EP), Disease: Intellectual disability (ID), GO biological process (BP): central nervous system development (CNS) and GO BP: Neurogenesis.

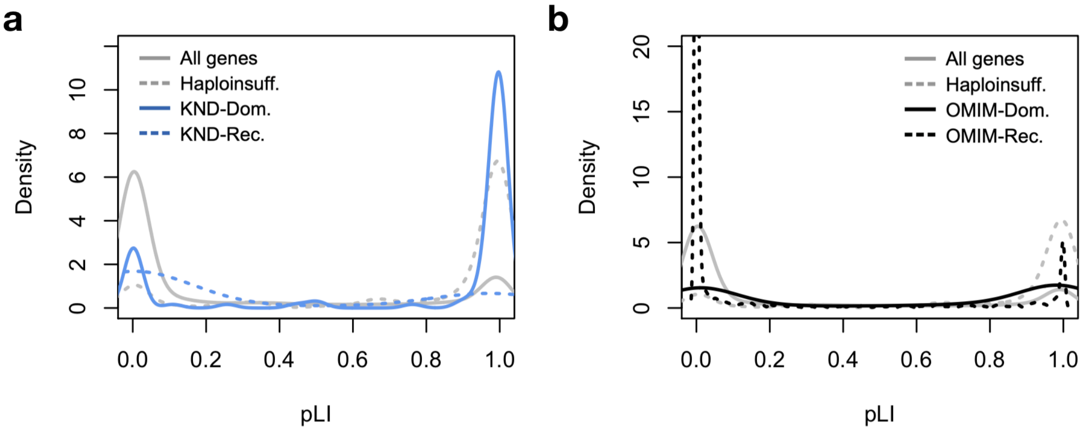

Figure S7. Distribution of pLI values.

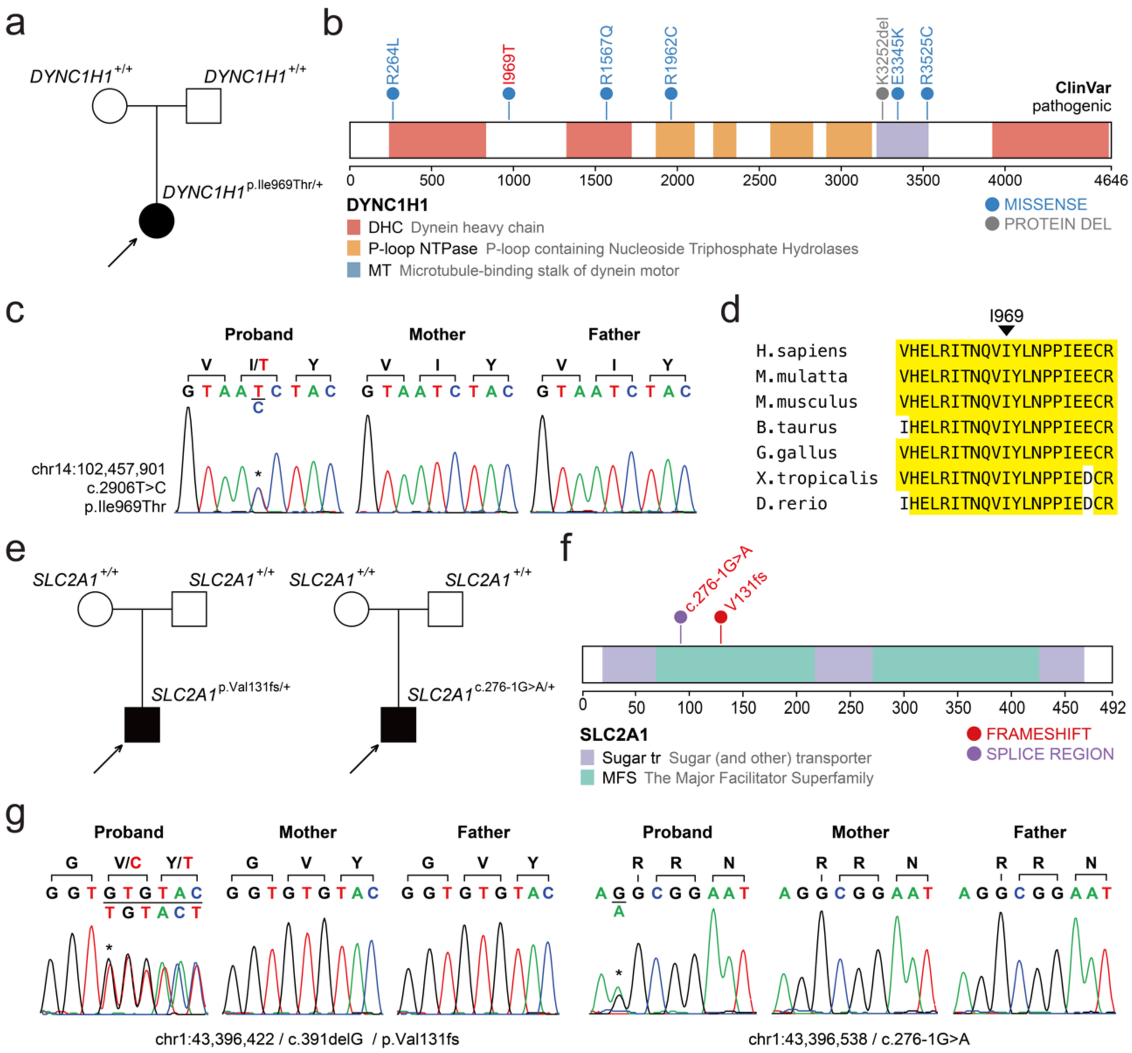

Figure S8. Presentation and validation of cases that WES-based analysis altered clinical courses. (a) A *de novo* variant in *DYNC1H1* identified from a patient with delayed development and hypotonia. (b) Pathogenic variants from ClinVar and domains of DYNC1H1 are displayed. A variant discovered in this study is shown in red. (c) Sanger traces validating the p.Ile969Thr variant. (d) Evolutionary conservation of the Ile969 residue across orthologs from major vertebrate species. (e) Two patients with dystonia and delayed motor development harbored LoF *de novo* variants in *SLC2A1*. (f) The variants found

in two patients and domains of SLC2A1 are displayed. (g) Sanger traces validating the *de novo* variants.

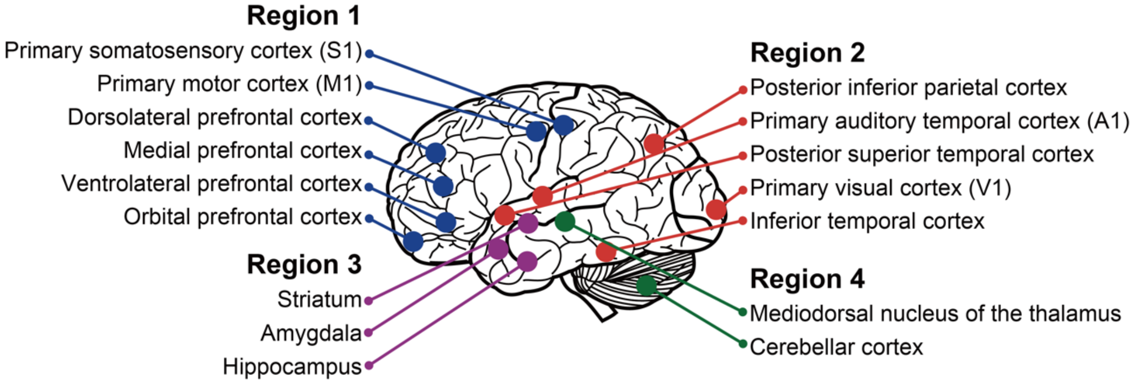

Figure S9. Anatomical components of each brain regions that were used for novel gene network analysis (modified from James.mcd.nz [CC BY-SA 4.0 (https://creativecommons.org/licenses/by-sa/4.0)]).

Table S1. List of copy number variations discovered in this study.

No.	Simple Diagnosis	CNV interval (Mb, hg19)	CNV length	Del/Dup	inheritance	No. of genes*	Notable gene(s) in the interval	Ref
**1**	Rett syndrome-like	chr1:0.6-3.1	2.6 Mb	Del	de novo	60	*MMP23B, GABRD, SKI, PRDM16*	8
**2**	Rett syndrome-like	chr1:107.0-113.1	6.1 Mb	Del	de novo	69	*WNT2B, NTNG1*	9
**3**	Hereditary spastic paraplegia	chr2:32.3-32.5	165.5 kb	Del	Inherited	3	*SPAST*	10
**4**	Epileptic encephalopathy	chr2:171.2-175.1	3.8 Mb	Del	de novo	18	*GAD1*	11
**5**	Unknown encephalopathy	chr2:234.8-242.8	8.0 Mb	Del	de novo	61	*HDAC4, PRLH, PER2, TWIST2, CAPN10, KIF1A, FARP2, D2HGDH, PDCD1*	12,13
**6**	Rett syndrome-like	chr3:9.0-13.0	4.0 Mb	Del	de novo	47	*SETD5, SLC6A1, SLC6A11*	14,15
**7**	Developmental delay with facial dysmorphism	chr3:44.2-48.0	3.9 Mb	Del	de novo	55	*SETD2, CSPG5, PTH1R, SMARCC1*	16

**8**	Rett syndrome-like	chr4:0.0-2.8	2.8 Mb	Del	de novo	42	*PIGG, CPLX2, FGFRL1, CTBP1, SLBP, LETM1*	17
**9**	Congenital hypotonia	chr5:139.0-139.6	567.7 kb	Del	de novo	7	*NRG2, PURA*	18
**10**	Epileptic encephalopathy	chr7:119.7-119.9	171.3 kb	Dup	de novo	0	*KCND2* upstream	19
**11**	Epileptic encephalopathy	chr9:0-47.3	47.3 Mb	Dup	de novo	202	n.d.	20
**12**	Developmental delay with facial dysmorphism	chr9:139.6-141.1	1.6 Mb	Del	de novo	56	*EHMT1*	21
**13**	Developmental delay with facial dysmorphism	chr12:53.6-54.1	537.9 kb	Dup	de novo	18	*AAAS, AMHR2, RARG*	22
**14**	Developmental delay, Joubert syndrome	chr15:20.3-23.7	3.4 Mb	Del	de novo	11	*TUBGCP5, NIPA1, NIPA2, CYFIP1*	23
**15**	Intellectual disability, facial dysmorphism	chr16:29.6-30.2	635.4 kb	Del	de novo	26	*MVP, CDIPT1, SEZ6L2, ASPHD1*	24
**16**	Global Developmental delay	chr18:52.5-53.3	787.2 kb	Del	de novo	3	*TCF4*	25
**17**	Hereditary spastic paraplegia	chr22:21.1-21.6	508.7 kb	Del	de novo	10	*CRKL*	26

**18**	Unknown severe retardation	chr22:42.8-51.3	8.6 Mb	Del	de novo	81	*SHANK3, IGF1*	27
**19**	Developmental delay, microcephaly	chrX::41.4-41.7	299.5 kb	Del	de novo	3	*CASK*	28
**20**	Unknown encephalopathy	chrX::100.1-107.3	7.2 Mb	Dup	hemizygous	76	*PLP1*	29
**21**	Neurodegeneration with severe motor developmental arrest	chrX:102.5-103.2	692.2 kb	Dup	de novo	15	*PLP1*	30
**22**	Epileptic encephalopathy	chrX:152.8-153.4	620.3 kb	Dup	de novo	21	*MECP2*	31
**23**	Hereditary spastic paraplegia	chrX:153.6-153.8	203.2 kb	Dup	hemizygous	15	*RPL10, ATP6P1, GDI1, IKBKG*	32,33

* Based on NCBI RefSeq database. Only protein-coding genes were counted.

Table S2. List of known neurologic disorder associated genes

Inheritance pattern	Gene	No.
Autosomal-dominant	*ACOX1, ACTB, ACTG1, ADCY5, ANKRD11, ANO3, ARID1B(3), ARID2, ASXL3(4), ATL1(4), ATP1A3, BICD2, BPTF(2), C19orf12, CHD3, CHD7, CLTC, COL1A1(2), CTNNB1(5), DHX30, DNM1(2), DNM1L, DYNC1H1(2), EHMT1, FOXG1(2), FOXP1, GABBR2, GABRB1, GFAP, GNAO1(5), GNB1, GRIA2, GRIN1(3), GRIN2A, GRIN2B(3), HECW2, ITPR1(3), KAT6A(2), KCNC1, KCNQ2, KIF1A(3), KMT2A, LRP5, MED13L, MRAS, MYH7, NALCN(2), NFIX, NFKB2, NIPBL, NRAS, PIK3CA(3), PMP22, POGZ, PPP2R5D(2), PPP3CA, PTEN, PURA(2), RAB11B, RARB(3), REEP1(2), RHOBTB2(2), SATB2, SCN1A, SCN2A(3), SCN4A, SETD2, SFTPC, SLC2A1(2), SLC6A1, SLC6A5, SMARCB1, SOX10, SOX5, SPAST, SPTBN2, STAT3, STXBP12(2), SYNGAP1, TCF4(3), TUBB3, TUBB4A(4), UBE3A, WASHC5, WDFY3, ZEB2*	86
Autosomal-recessive	*ABAT, ALDH7A1, APTX, ARSA(2), ASAH1, ATAD3A, ATP6V0A2, BRAT1(2), CC2D2A, CLN6(2), CNTNAP1, CWF19L1, CYP7B1, DEGS1, ECHS1(2), EIF2B2, EIF2B3, ERCC5, FIG4, GCDH, GDAP1, GLB1(2), GMPPB(2), HADHA, HSD17B4, IARS2(2), IGHMBP2, KIAA1109, KIF1A,*	55

	KLHL40, LONP1(2), MICU1, NARS2(2), NDUFAF6(2), NDUFS1, NDUFV1, PEX5, PIGN, PLA2G6(2), PMM2, PNPT1(2), POLR1C, PPT1, PRUNE1, RYR1, SACS(2), SLC19A3(2), SLC25A15, ST3GAL5, SURF1, THOC6, TMEM173, TUBA8, WDR62, WDR81	
X-linked	*ATRX(3), CASK(2), CDKL5(3), EDA, EIF2S3, GRIA3(2), HDAC8, HPRT1, IQSEC2(2), MECP2(3), MED12, NAA10, NSDHL, OPHN1(2), PAK3, PCDH19, PDHA1(2), PHF6, PLP1, SLC16A2, SMC1A(2), USP9X, ZC4H2(4)*	23

( ): Number of patients (more than one patient only)

References

1. Lee, J. S. *et al.* GM3 synthase deficiency due to ST3GAL5 variants in two Korean female siblings: Masquerading as Rett syndrome-like phenotype. *Am. J. Med. Genet. Part A* **170**, 2200–2205 (2016).
2. Boccuto, L. *et al.* A mutation in a ganglioside biosynthetic enzyme, ST3GAL5, results in salt & pepper syndrome, a neurocutaneous disorder with altered glycolipid and glycoprotein glycosylation. *Hum. Mol. Genet.* **23**, 418–433 (2014).
3. Strickland, A. V. *et al.* Mutation screen reveals novel variants and expands the phenotypes associated with DYNC1H1. *J. Neurol.* **262**, 2124–2134 (2015).
4. Kim, S. Y. *et al.* Atypical presentation of infantile-onset farber disease with novel ASAH1 mutations. *Am. J. Med. Genet. Part A* **170**, 3023–3027 (2016).
5. Hully, M. *et al.* From splitting GLUT1 deficiency syndromes to overlapping phenotypes. *Eur. J. Med. Genet.* **58**, 443–454 (2015).
6. Lamers, I. J. C. *et al.* Recurrent De Novo Mutations Disturbing the GTP/GDP Binding Pocket of RAB11B Cause Intellectual Disability and a Distinctive Brain Phenotype. *Am. J. Hum. Genet.* **101**, 824–832 (2017).
7. Sobreira, N., Schiettecatte, F., Valle, D. & Hamosh, A. GeneMatcher: A Matching Tool for Connecting Investigators with an Interest in the Same Gene. *Hum. Mutat.* **36**, 928–930 (2015).
8. Jordan, V. K., Zaveri, H. P. & Scott, D. A. 1p36 deletion syndrome: An update. *Appl. Clin. Genet.* **8**, 189–200 (2015).
9. Bisgaard, A.-M., Rasmussen, L. N., Moller, H. U., Kirchhoff, M. & Bryndorf, T. Interstitial deletion of the short arm of chromosome 1 (1p13.1p21.1) in a girl with mental retardation, short stature and colobomata. *Clin. Dysmorphol.* **16**, 109–112 (2007).

10. Boone, P. M. *et al.* The alu-rich genomic architecture of SPAST predisposes to diverse and functionally distinct disease-associated CNV alleles. *Am. J. Hum. Genet.* **95**, 143–161 (2014).

11. Bharadwaj, R. *et al.* Conserved Chromosome 2q31 Conformations Are Associated with Transcriptional Regulation of GAD1 GABA Synthesis Enzyme and Altered in Prefrontal Cortex of Subjects with Schizophrenia. *J. Neurosci.* **33**, 11839–11851 (2013).

12. Leroy, C. *et al.* The 2q37-deletion syndrome: an update of the clinical spectrum including overweight, brachydactyly and behavioural features in 14 new patients. *Eur. J. Hum. Genet.* **21**, 602–612 (2013).

13. Williams, S. R. *et al.* Haploinsufficiency of HDAC4 causes brachydactyly mental retardation syndrome, with brachydactyly type E, developmental delays, and behavioral problems. *Am. J. Hum. Genet.* **87**, 219–228 (2010).

14. Kuechler, A. *et al.* Loss-of-function variants of SETD5 cause intellectual disability and the core phenotype of microdeletion 3p25.3 syndrome. *Eur. J. Hum. Genet.* **23**, 753–760 (2015).

15. Dikow, N. *et al.* 3p25.3 microdeletion of GABA transporters SLC6A1 and SLC6A11 results in intellectual disability, epilepsy and stereotypic behavior. *Am. J. Med. Genet. Part A* **164**, 3061–3068 (2014).

16. Lovrecic, L., Bertok, S. & Žerjav Tanšek, M. A New Case of an Extremely Rare 3p21.31 Interstitial Deletion. *Mol. Syndromol.* **7**, 93–98 (2016).

17. Battaglia, A., Carey, J. C. & South, S. T. Wolf-Hirschhorn syndrome: A review and update. *Am. J. Med. Genet. Part C Semin. Med. Genet.* **169**, 216–223 (2015).

18. Hosoki, K. *et al.* Clinical phenotype and candidate genes for the 5q31.3

microdeletion syndrome. *Am. J. Med. Genet. Part A* **158 A**, 1891–1896 (2012).

19. Zhao, J., Noon, S. E., Krantz, I. D. & Wu, Y. A de novo interstitial deletion of 7q31.2q31.31 identified in a girl with developmental delay and hearing loss. *Am. J. Med. Genet. Part C Semin. Med. Genet.* **172**, 102–108 (2016).
20. Abu-Amero, K. K. *et al.* A de novo marker chromosome derived from 9p in a patient with 9p partial duplication syndrome and autism features: genotype-phenotype correlation. *BMC Med. Genet.* **11**, 135 (2010).
21. Kleefstra, T., Nillesen, W. M. & Yntema, H. G. *Kleefstra Syndrome. GeneReviews®* (1993).
22. Jonsson, D. I. *et al.* A de novo 1.13 Mb microdeletion in 12q13.13 associated with congenital distal arthrogryposis, intellectual disability and mild dysmorphism. *Eur. J. Med. Genet.* **55**, 437–440 (2012).
23. Doornbos, M. *et al.* Nine patients with a microdeletion 15q11.2 between breakpoints 1 and 2 of the Prader-Willi critical region, possibly associated with behavioural disturbances. *Eur. J. Med. Genet.* **52**, 108–115 (2009).
24. Crepel, A. *et al.* Narrowing the critical deletion region for autism spectrum disorders on 16p11.2. *Am. J. Med. Genet. Part B Neuropsychiatr. Genet.* **156**, 243–245 (2011).
25. Rosenfeld, J. A. *et al.* Genotype-phenotype analysis of TCF4 mutations causing Pitt-Hopkins syndrome shows increased seizure activity with missense mutations. *Genet. Med.* **11**, 797–805 (2009).
26. Burnside, R. D. 22q11.21 deletion syndromes: A review of proximal, central, and distal deletions and their associated features. *Cytogenetic and Genome Research* **146**, 89–99 (2015).
27. Shcheglovitov, A. *et al.* SHANK3 and IGF1 restore synaptic deficits in neurons

from 22q13 deletion syndrome patients. *Nature* **503**, 267–271 (2013).

28. Atasoy, D. *et al.* Deletion of CASK in mice is lethal and impairs synaptic function. *Proc. Natl. Acad. Sci.* **104**, 2525–2530 (2007).

29. Woodward, K., Kendall, E., Vetrie, D. & Malcolm, S. Pelizaeus-Merzbacher disease: identification of Xq22 proteolipid-protein duplications and characterization of breakpoints by interphase FISH. *Am J Hum.Genet.* **63**, 207–217 (1998).

30. Shimojima, K. *et al.* Reduced PLP1 expression in induced pluripotent stem cells derived from a Pelizaeus-Merzbacher disease patient with a partial PLP1 duplication. *J. Hum. Genet.* **57**, 580–586 (2012).

31. Van Esch, H. *et al.* Duplication of the MECP2 region is a frequent cause of severe mental retardation and progressive neurological symptoms in males. *Am. J. Hum. Genet.* **77**, 442–53 (2005).

32. Vandewalle, J. *et al.* Dosage-Dependent Severity of the Phenotype in Patients with Mental Retardation Due to a Recurrent Copy-Number Gain at Xq28 Mediated by an Unusual Recombination. *Am. J. Hum. Genet.* **85**, 809–822 (2009).

33. Fusco, F., D'Urso, M., Miano, M. G. & Ursini, M. V. The LCR at the IKBKG Locus Is Prone to Recombine. *Am. J. Hum. Genet.* **86**, 650–652 (2010).
